# Association between long working hours and unmet dental needs in wage workers

**DOI:** 10.1186/s12903-023-03289-0

**Published:** 2023-08-13

**Authors:** Youngjin Choi, Inah Kim, Jaechul Song

**Affiliations:** 1 Department of Public Health Sciences, Hanyang University Graduate School, Seoul, South Korea; 2 Hanyang University Graduate School of Public Health, Seoul, South Korea; 3 Department of Occupational and Environmental Medicine, Hanyang University Medical Center, Seoul, South Korea

**Keywords:** Health services accessibility, Health services needs and demand*, Dental health services, Occupational health, Time factors, Work schedule tolerance*

## Abstract

**Background:**

Many previous studies on the reasons behind unmet dental needs focus on economic issues. However, in this research, we aimed to investigate the relationship between long working hours and unmet dental needs while considering the influence of occupational factors in wage workers.

**Methods:**

This study used data from the Korea National Health and Nutrition Examination Survey (2012–2018) and analyzed a sample of 12,104 wage workers. Unmet dental needs were defined as cases in which individuals did not receive dental care, despite their need for examination or treatment, within the last year. Long working hours were defined as exceeding 52 h per week, based on the standard working hours stipulated by the Labor Standards Act. A binomial model was applied to calculate the prevalence ratio through multivariate logistic regression analysis.

**Results:**

The prevalence of unmet dental needs was observed in 3,948 cases (32.5%), among which 1,478 attributed their presence to lack of time. The prevalence of unmet dental needs showed an inverse relationship with the education level and household income. The wage workers who worked long hours had the highest prevalence of unmet dental needs. Long working hours were found to be 1.18 times (95% CI 1.07–1.29) more likely to result in unmet dental care compared to working less than 40 h. The relationship between long working hours and unmet dental needs were statistically significant only in men (PR 1.24, 95% CI 1.07–1.43). However, the relationship between long working hours and unmet dental needs owing to time were in both men and women (men: PR 1.59, 95% CI 1.20–2.11, women: PR 1.90, 95% CI 1.48–2.43).

**Conclusions:**

This study confirmed that long working hours and unmet dental needs are related when occupational factors are taken into consideration, despite the absence of oral health indicators. Using this study as a reference, further research is necessary to identify the underlying causes of unmet dental care and to improve access to dental services in the future.

**Supplementary Information:**

The online version contains supplementary material available at 10.1186/s12903-023-03289-0.

## Background

Oral health is crucial for a healthy and high-quality life, particularly in an aging society. Given the high incidence and prevalence of oral diseases, their management and prevention are of utmost importance. However, individuals living in poor socioeconomic environments often encounter difficulties in accessing adequate oral healthcare, which may impede their ability to maintain good oral health [1, 2].

South Korea has expanded health insurance coverage to reduce the financial burden of dental treatments, allowing for greater access to oral health care services [3, 4]. Despite this expansion, according to a Report from the Korea National Health and Nutrition Examination Survey (KNHANES) of Dental Health Care Utilization [5] during the period from 2017 through 2020, the prevalence in the country remains high, at 30.2 ~ 40.1% comparing to Medical Health Care Utilization. The leading reason behind unmet dental needs is lack of time during the period from 2017 ~ 2020 compared with economic reasons.

A relationship has been established between long working hours and unmet medical needs [6, 7]. As reported to the Organization for Economic Co-operation and Development (OECD), South Korea has an annual average of 1,915 working hours, which is longer than the average of OECD countries (1,716 h) or Japan (1,607 h). Previous studies have shown that long working hours have a negative impact on the physical and mental health of wage workers [8–10].

However, research on unmet dental care has predominantly focused on economic factors [11–13], while studies examining occupational factors and the relationship between unmet dental care and long working hours are lacking. Therefore, in this study, we aimed to investigate the association between long working hours and unmet dental needs among wage workers, taking into consideration various occupational factors.

## Methods

### Study population and data collection

In this study, we used data from the Korea National Health and Nutrition Examination Survey (KNHANES), conducted by the Korea Centers for Disease Control and Prevention (KCDC) between 2012 and 2018. The data was collected using a rotational sample survey with a multi-stage clustered probability design, representing the entire population. The survey consisted of health interviews, physical examinations, and nutrition surveys [14]. This study examined the relationship between long working hours and unmet dental care among wage workers, using data from the health interview survey.

**Fig. 1:**
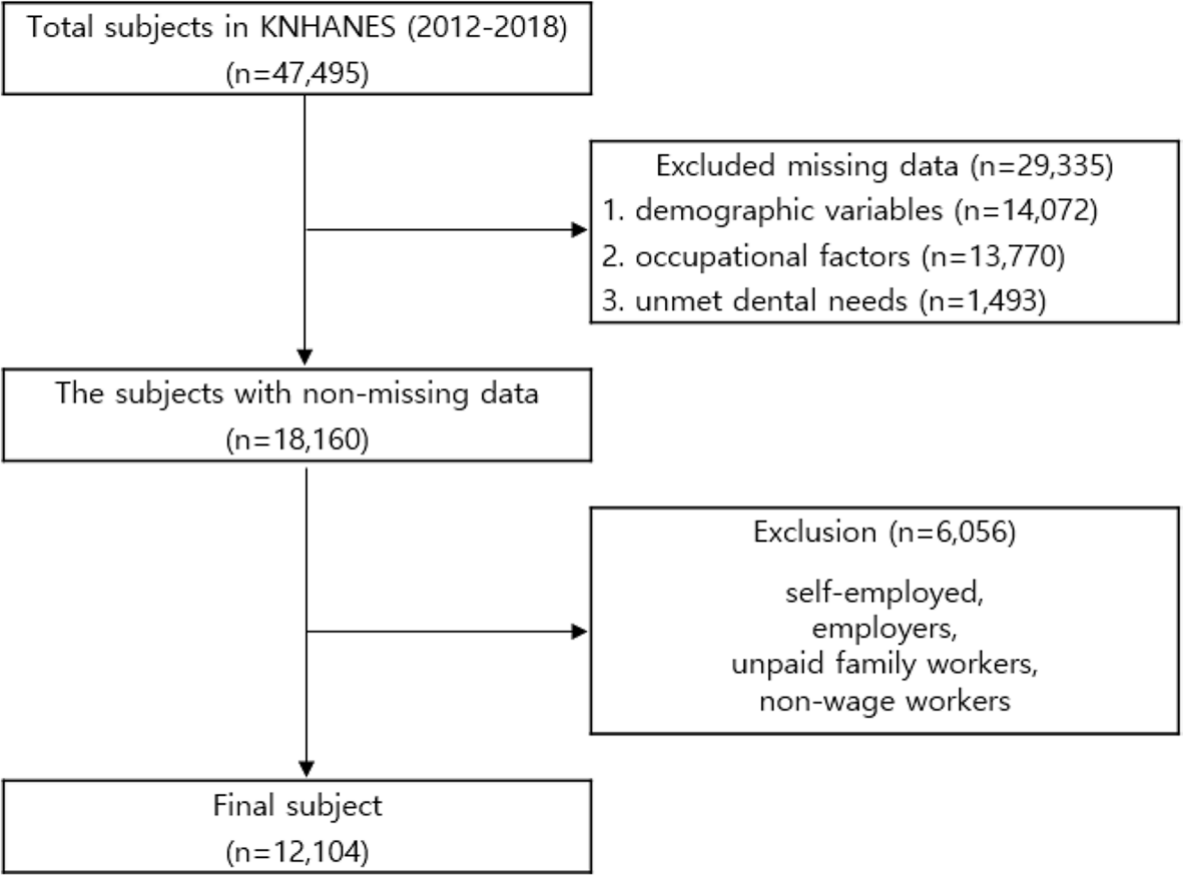
Target selection process flowchart

The database for this study included the basic and the oral databases from 2012 to 2018. The resulting dataset included 47,495 subjects, out of which 29,335 were excluded due to missing data on demographic variables, occupational factors (including long working hours), or unmet dental needs. The study subjects were defined as economically active individuals working for wages. Therefore, 6,056 subjects—self-employed, employers, unpaid family workers, and non-wage workers—were excluded, resulting in a final number of 12,104 subjects (Fig. 1).

### Measurement and definition of variables

#### Dependent variable

The dependent variable in this study was “unmet dental needs,” defined as the absence of dental care, despite the recognition of its necessity, within the past year. The primary reasons for unmet dental needs were classified into economic, time-related, or other. Economic reasons were indicated by a single response of “economic reasons”. Time-related reasons were indicated by responses such as “because the dental clinic was not open when I wanted to go”, “because I was unable to leave my workplace”, and “because I had to take care of my child”. If the response did not fall into either the economic or time-related categories, it was classified as other [15, 16].

#### Independent variable

The independent variable in this study was “long working hours”, which was measured as the number of working hours per week, excluding mealtime. Working hours were categorized according to the Labor Standards Act of the South Korea based on standard working hours, and long working hours were defined as any hours worked beyond the standard 52 h per week, including overtime.

#### Confounding variables

The study identified confounding variables as demographic variables and occupational factors. The demographic variables comprised gender, age, marital status, household income level, education level, and residential area. Age was classified into three groups: 19–39 years, 40–59 years, and 60 years or older. Regarding residential areas, the surveyed data were used to classify administrative districts into three types based on population distribution. In the data, *dong* refers to urban areas with a relatively large population distribution, while *eup/myeon* refer to rural areas. The occupational factors consisted of occupational group, employment status, and work schedule. The occupational groups were re-categorized into three groups based on the South Korean Standard Classification of Occupations of the National Statistical, namely white, pink, and blue-collar workers, as done in previous studies [17–19]. White collars included managers, experts, related workers, and office workers. Pink collars included only service and sales workers, while blue collars consisted of skilled workers in agriculture, forestry and fisheries, technicians, machinery operation and assembly workers, and simple labor workers. Employment status was distinguished based on whether the workers worked full-time or not, the latter being classified as temporary or daily workers. The work schedule was categorized into three groups: daily work (DW), rotating shift work (RSW), and others, with RSW being operationally defined as day-night regular and 24-h shift work (SW) [20], while others included two or more between split work, evening work, night work, and irregular SW.

### Statistical analysis

The KNHANES data is a survey with a complex sampling design that uses the stratified cluster sampling method. According to the KNHANES data analysis guide, it is necessary to consider the weights variable when using the relevant data. The weights variable includes two types: household unit weights and personal unit weights. This study defines the study subjects as economically active individuals working for wages. Therefore, personal unit weights were employed in the analysis to account for the sampling design and accurately represent the target population [14, 21]. Regarding the 7th period (2016–2018) database, some of the survey areas are extracted. Therefore, because the sample size of the data is small compared with other periods, the integrated weight separately provided by the KNHANES was applied. The prevalence of unmet dental needs was found to be 32.5%. However, using logistic regression analysis may result in a risk overestimation when the prevalence of a disease exceeds 10% [22, 23]. Therefore, we calculated the prevalence ratio (PR) through multivariate logistic regression analysis using a binomial model. The statistical software SAS Version 9.4 (SAS Institute Inc., Cary, NC, USA) was used for analysis, and the level of statistical significance was established at 0.05.

## Results

### Differences in unmet dental needs

Table 1 presents the characteristics of the final sample of study subjects. A total of 3,948 (32.5%) subjects reported unmet dental needs, with 1,478 (37.4%) citing time as the primary reason and 1,112 (28.2%) citing economic reasons. The unmet dental needs were found to be higher among women than men and they decreased with the increase in the education or household income levels. Furthermore, regarding working hours, the prevalence of unmet dental needs was highest among individuals working 52 h, followed by those working 40 h, and then 40 ~ 52 h. White-collar workers had the lowest prevalence of unmet dental needs based on occupational group, while full-time workers had a higher prevalence of unmet dental needs compared to non-full-time wage workers.

**Table 1:** Characteristics of study subjects

		Total	No		Yes		*p*
			N	wt%	N	wt%	
Gender	Man	5963	4177	70.0	1786	30.0	< .0001
	Woman	6141	3979	64.8	2162	35.2	
Age group (years)	20 ~ 39	4646	3128	67.9	1518	32.1	.256
	40 ~ 59	5482	3668	66.7	1814	33.3	
	≥ 60	1976	1360	69.2	616	30.8	
Marital status	Experience	9541	6435	67.5	3106	32.5	.028
	In-Experience	2563	1721	67.7	842	32.3	
Education level	≤ Middle school	2394	1513	63.5	881	36.5	< .0001
	High school	4195	2723	64.6	1472	35.4	
	≥ University	5515	3920	71.2	1595	28.8	
Household income	Under	1137	696	61.5	441	38.5	< .0001
	Medium low	2845	1813	63.4	1032	36.6	
	Slander	3832	2555	66.8	1277	33.2	
	Award	4290	3092	72.3	1198	27.7	
Residence	Dong	10324	7029	68.3	3295	31.7	.001
	Eup, Myeon	1780	1127	62.5	653	37.5	
Occupational group	White collar	5680	3965	70.3	1715	29.8	< .0001
	Pink collar	2093	1374	66.5	719	33.5	
	Blue collar	4331	2817	64.3	1514	35.7	
Working hours (per week)	< 40 h	4124	2770	67.0	1354	33.0	.002
	40 ~ 52 h	5875	4022	69.0	1853	31.0	
	≥ 52 h	2105	1364	64.3	741	35.7	
Employment status	Full-time	8429	5816	69.2	2613	30.8	< 0001
	Temporary/Daily	3675	2340	63.6	1335	36.4	
Work schedule	Day work	10012	6732	67.3	3280	32.7	.042
	Rotational shift work	784	565	72.2	219	27.8	
	Other	1308	859	66.7	449	33.3	
Sum		12104	8156	67.5	3948	32.5	

### Relationship between long working hours and unmet dental needs

Table 2 shows the results of the analysis conducted to investigate the relationship between long working hours and unmet dental needs. The prevalence of unmet dental needs was found to be 1.18 times higher in women than in men (95% CI 1.11–1.26) and more common among younger individuals and those with lower income levels. Moreover, individuals who worked long hours were 1.18 times more likely to experience unmet dental needs compared to those who worked less than 40 h (95% CI 1.07–1.29). However, long working hours were only found to be statistically significant in the relationship with unmet dental needs in men (PR 1.24, 95% CI 1.07–1.43). In terms of work schedule, the RSW had a statistically significant 0.75 times lower prevalence of unmet dental needs compared to DW, but only in men (95% CI 0.63–0.90).

**Table 2:** Relationship between long working hours and unmet dental needs (Adj-PR, *N* = 12,104)

		Total		Men		Women	
		Adj-PR	95% CI	Adj-PR	95% CI	Adj-PR	95% CI
Gender	Man	1.00					
	Woman	1.18	(1.11–1.26)				
Age group (y)	20 ~ 39	1.26	(1.11–1.42)	1.21	(1.01–1.45)	1.33	(1.13–1.58)
	40 ~ 59	1.24	(1.12–1.37)	1.28	(1.09–1.49)	1.25	(1.09–1.43)
	≥ 60	1.00		1.00		1.00	
Marital status	Experience	1.01	(0.92–1.10)	1.03	(0.90–1.18)	1.02	(0.90–1.15)
	In-Experience	1.00		1.00		1.00	
Education level	≤ Middle school	1.15	(1.03–1.29)	0.99	(0.83–1.18)	1.28	(1.10–1.49)
	High school	1.14	(1.05–1.23)	1.21	(1.09–1.36)	1.08	(0.97–1.21)
	≥ University	1.00		1.00		1.00	
Household income	Under	1.30	(1.16–1.47)	1.26	(1.03–1.54)	1.30	(1.13–1.51)
	Medium low	1.23	(1.14–1.34)	1.22	(1.07–1.38)	1.25	(1.12–1.40)
	Slander	1.14	(1.06–1.24)	1.18	(1.05–1.32)	1.11	(1.00–1.23)
	Award	1.00		1.00		1.00	
Residence	Dong	1.00		1.00		1.00	
	Eup, Myeon	1.13	(1.04–1.22)	1.12	(0.99–1.26)	1.13	(1.01–1.26)
Occupational group	White collar	1.00		1.00		1.00	
	Pink collar	0.97	(0.89–1.07)	1.00	(0.85–1.18)	0.96	(0.85–1.08)
	Blue collar	1.06	(0.97–1.16)	1.09	(0.97–1.23)	1.02	(0.89–1.16)
Working hours (per week)	< 40 h	1.00		1.00		1.00	
	40 ~ 52 h	1.04	(0.97–1.13)	1.04	(0.91–1.19)	1.05	(0.96–1.15)
	≥ 52 h	1.18	(1.07–1.29)	1.24	(1.07–1.43)	1.08	(0.94–1.23)
Employment status	Full-time	1.00		1.00		1.00	
	Temporary/Daily	1.07	(0.99–1.15)	1.09	(0.97–1.24)	1.08	(0.98–1.19)
Work schedule	Day work	1.00		1.00		1.00	
	Rotational shift work	0.86	(0.74–0.99)	0.75	(0.63–0.90)	1.12	(0.90–1.40)
	Other	0.97	(0.88–1.07)	1.00	(0.85–1.18)	0.95	(0.84–1.08)

### Relationship between long working hours and unmet dental needs due to time

Table 3 presents the results of the analysis conducted to investigate the relationship between long working hours and unmet dental needs due to lack of time. The table provides the PR for 1,478 subjects who reported unmet dental needs due to lack of time compared with 8,156 subjects who did not report them.

**Table 3:** Relationship between long working hours and unmet dental needs due to time (Adj-PR, *N* = 9,634)

		Total		Men		Women	
		Adj-PR	95% CI	Adj-PR	95% CI	Adj-PR	95% CI
Gender	Man	1.00					
	Woman	1.20	(1.07–1.34)				
Age group (y)	20 ~ 39	1.45	(1.12–1.87)	1.39	(0.98–1.97)	1.62	(1.11–2.36)
	40 ~ 59	1.22	(0.97–1.53)	1.33	(0.97–1.82)	1.18	(0.85–1.65)
	≥ 60	1.00		1.00		1.00	
Marital status	Experience	0.93	(0.80–1.07)	0.98	(0.79–1.22)	0.94	(0.77–1.15)
	In-Experience	1.00		1.00		1.00	
Education level	≤ Middle school	1.11	(0.89–1.40)	0.78	(0.56–1.09)	1.67	(1.20–2.33)
	High school	1.16	(1.01–1.33)	1.15	(0.95–1.39)	1.22	(1.00–1.48)
	≥ University	1.00		1.00		1.00	
Household income	Under	1.01	(0.77–1.33)	0.88	(0.55–1.39)	1.08	(0.76–1.53)
	Medium low	1.04	(0.90–1.21)	0.97	(0.78–1.20)	1.13	(0.92–1.39)
	Slander	1.12	(0.98–1.27)	1.11	(0.93–1.33)	1.10	(0.92–1.32)
	Award	1.00		1.00		1.00	
Residence	Dong	1.00		1.00		1.00	
	Eup, Myeon	1.22	(1.05–1.42)	1.20	(0.97–1.47)	1.23	(1.00–1.51)
Occupational group	White collar	1.00		1.00		1.00	
	Pink collar	0.85	(0.71–1.03)	1.06	(0.80–1.41)	0.69	(0.54–0.88)
	Blue collar	0.94	(0.80–1.10)	1.10	(0.89–1.35)	0.73	(0.56–0.95)
Working hours (per week)	< 40 h	1.00		1.00		1.00	
	40 ~ 52 h	1.36	(1.16–1.59)	1.08	(0.83–1.42)	1.52	(1.26–1.84)
	≥ 52 h	1.86	(1.55–2.23)	1.59	(1.20–2.11)	1.90	(1.48–2.43)
Employment status	Full-time	1.00		1.00		1.00	
	Temporary/Daily	0.92	(0.79–1.07)	0.88	(0.69–1.13)	1.00	(0.83–1.22)
Work schedule	Day work	1.00		1.00		1.00	
	Rotational shift work	0.69	(0.53–0.90)	0.51	(0.36–0.72)	1.23	(0.82–1.82)
	Other	0.70	(0.56–0.88)	0.64	(0.44–0.93)	0.75	(0.56–1.01)

The findings suggest that men who worked more than 52 h had a higher prevalence of unmet dental needs owing to a lack of time compared with those who worked less than 40 h, with a PR of 1.59 (95%CI: 1.20–2.11). Similarly, women also showed a significant relationship between unmet dental needs owing to a lack of time and working 52 h, with a PR of 1.90 (95% CI: 1.48–2.43). Regarding the work schedule, the RSW was associated with a 0.69 times lower prevalence of unmet dental needs compared to DW (95% CI 0.53–0.90). However, this association was statistically significant only in male workers, with a PR of 0.51 (95% CI 0.36–0.72).

## Discussion

This study aimed to investigate the relationship between long working hours and unmet dental needs, with a sub-analysis focusing on this relationship due to lack of time in wage workers. Data used in the analysis were collected from the KNHANES conducted between 2012 and 2018. The prevalence of unmet dental needs among the final sample was 32.5%. Previous studies on unmet dental needs have typically reported odds ratio (OR) [24–26]. However, it is known that the OR may be overestimated if the prevalence of the disease exceeds 10%. Therefore, in this study, we calculated the PR taking into account these considerations and the fact that the data used were cross-sectional.

Results showed that the prevalence of unmet dental needs was higher among individuals with lower levels of education, household income. Additionally, the prevalence of unmet dental needs was found to be higher in *eup/myeon* areas than in *dong* areas. Regarding the occupational group, blue, pink and white collar were the highest, in that order. Long working hours showed a higher prevalence of unmet dental needs, particularly due to lack of time (35.7% prevalence rate). However, for men, individuals who worked more than 52 h were 1.24 times more likely to have unmet dental needs than those who worked less than 40 h. Moreover, unmet dental needs due to lack of time were higher in both men and women who worked more than 52 h, and those working less than 40 h.

Previous studies have reported economic reasons as having a significant impact on unmet dental needs [26, 27]. Similarly, this study found that as the household income decreased, the likelihood of unmet dental needs increased. Notably, income disparities in access to dental care are not limited to wage workers [4]. In particular, the high cost of dental treatment can be a factor that limits access to dental healthcare among individuals with low income [7, 28, 29].

In domestic studies, the relationship between long working hours and unmet medical needs appeared to be consistent regardless of income level. In particular, individuals in the low-income group who worked long hours were more likely to experience unmet medical needs due to lack of time [30]. Similarly, working long hours posed a higher risk for unmet dental needs than working 40 to 52 h [31]. In this study as well, the long working hours presented a significant risk of unmet dental needs owing to lack of time. Particularly, the risk was higher for women than men. According to reports of unmet dental needs due to lack of time were more prevalent among married women than among single women (*p* < 0.0001; Additional file 3). For women, conflict between work and housework increases with the increase in working hours [32]. Additionally, the Family and Childbirth Survey (2021) reported that on average, women spend over 5 h per week on parenting duties, while men spend approximately 2 h. Therefore, women with children may find it challenging to visit a dentist. However, in this study, unmet dental needs owing to lack of time were evaluated based on responses such as “because when I wanted [to go], it was not open”, “because I could not leave my workplace”, and “because I had to take care of my child”. Moreover, unmet dental needs owing to lack of time were 1.18 times higher for women than for men. The response "because I have to take care of my child" includes other factors, such as childcare. Therefore, when comparing men and women, it is necessary to be careful in interpretation considering the possibility that women may be overestimated.

In a study conducted by Ha et al., no significant relationship was found between SW and unmet medical needs [33]. By contrast, Lee et al. reported that unmet dental needs due to lack of time were higher among female shift workers compared to those engaged in DW. Interestingly, the current study revealed a lower prevalence of unmet dental needs among the RSW compared to DW in men. Despite the long working hours in SW, organized schedules in rotating shifts often provide access to medical services during days-off and holidays. However, it is possible that female shift workers may experience unmet needs during days off or holidays due to household and childcare responsibilities.

The inconsistent relationship between SW and unmet needs across studies can be attributed to differences in the definitions of SW. Ha et al. defined SW as night work, while Lee et al. included all non-DW. By contrast, the current study defined SW as including regular day, night, and 24-h shifts. Therefore, results should be interpreted cautiously. Notably, the rate of oral health examinations in Korea is only 32%, compared to a general health examination rate of 79%. This highlights a general lack of awareness regarding the importance of oral health management [34]. Furthermore, dental hospitals and clinics in South Korea typically operate from 10:00 am to 18:00 pm for treatment hours. Based on our research findings, it can be inferred that men who work during these hours may find it challenging to visit the dentist at a convenient time, as they would take time away from work.

This study has several limitations. First, because it used self-reported data, response bias might have occurred [35, 36]. Second, the analysis did not include any oral health indicators. Lastly, the available data sources were limited in terms of labor-related information. However, despite these limitations, our study found a significant relationship between long working hours and unmet dental needs. Particularly, unmet dental needs owing to lack of time were also significantly related to long working hours, higher than household level. Additionally, this study is noteworthy for calculating the PR while considering the scale of unmet dental needs.

## Conclusion

Despite the limitations of our data sources, we were able to confirm a significant relationship between long working hours and unmet dental needs, which may be due to a lack of time for oral examination or treatment. Notably, this lack of time for oral healthcare appears to differ by gender, and our findings suggest that male day workers may face difficulties accessing dental care. To improve medical accessibility and address unmet dental needs in the future, further research is needed to identify.

### Supplementary Information

**Additional file 1. **Distribution of research subjects by reason of unsatisfied dental care.

**Additional file 2. **Distribution of man study subjects by working hours.

**Additional file 3. **Distribution of woman study subjects by working hours.

## Data Availability

The raw data from KNHANES can be accessed at https://knhanes.kdca.go.kr/knhanes/sub03/sub03_02_05.do. The datasets generated and/or analyzed during this study are available upon request to the corresponding author, YJC, owing to the nature of the research and restrictions imposed by our IRB.

## Abbreviations

KNHANES: The Korea National Health and Nutrition Examination Survey
KCDC: The Korea Centers for Disease Control and Prevention
PR: Prevalence ratio
OR: Odds ratio
DW: Day work
RSW: Rotating shift work
SW: Shift work
