# Supplementary material for: Association between long working hours and unmet dental needs in wage workers
Source: BMC Oral Health. 2023 Aug 13;23:570. doi: 10.1186/s12903-023-03289-0 (PMC10424332; doi:10.1186/s12903-023-03289-0)
Supplement: Supplementary file 1 — Additional file 1. Distribution of research subjects by reason of unsatisfied dental care. [file 12903_2023_3289_MOESM1_ESM.docx]

Additional file 1: Distribution of research subjects by reason of unsatisfied dental care.

|  | | Economic issues | | Lack of time | | Other | |  |
| --- | --- | --- | --- | --- | --- | --- | --- | --- |
|  |  |  |  |  |  |  |  | *p* |
|  |  | N | wt% | N | wt% | N | wt% |  |
|  | Man | 449 | 24.4 | 736 | 41.8 | 601 | 33.8 | .0001 |
| Gender |  |  |  |  |  |  |  |  |
|  | Woman | 663 | 30.6 | 742 | 34.7 | 757 | 34.7 |  |
|  | 20~39 | 327 | 22.9 | 687 | 45.1 | 504 | 32.0 | .0001 |
| Age group  (y) | 40~59 | 517 | 29.2 | 636 | 34.4 | 661 | 36.4 |  |
|  | ≥ 60 | 268 | 38.7 | 155 | 27.0 | 193 | 34.2 |  |
|  | Experience | 912 | 28.8 | 1114 | 36.2 | 1080 | 34.9 | .001 |
| Marital status |  |  |  |  |  |  |  |  |
|  | In-Experience | 200 | 23.9 | 364 | 43.9 | 278 | 32.2 |  |
|  | ≤ Middle school | 418 | 45.8 | 226 | 26.3 | 237 | 27.9 | .0001 |
| Education  level | High school | 436 | 30.2 | 528 | 35.4 | 508 | 34.5 |  |
|  | ≥ University | 258 | 16.9 | 724 | 46.2 | 613 | 36.9 |  |
|  | Under | 244 | 51.2 | 86 | 22.0 | 111 | 26.8 | .0001 |
| Household income | Medium Low | 397 | 39.1 | 330 | 31.0 | 305 | 30.0 |  |
|  | Slander | 304 | 23.6 | 525 | 41.6 | 448 | 34.8 |  |
|  | Award | 167 | 14.4 | 537 | 45.8 | 494 | 39.7 |  |
|  | Dong | 928 | 27.7 | 1233 | 38.1 | 1134 | 34.1 | .920 |
| Residence |  |  |  |  |  |  |  |  |
|  | Eup, Myeon | 184 | 26.7 | 245 | 38.4 | 224 | 34.8 |  |
|  | White collar | 277 | 16.1 | 783 | 46.6 | 655 | 37.2 | .0001 |
| Occupational  group | Pink collar | 249 | 34.8 | 217 | 30.5 | 253 | 34.7 |  |
|  | Blue collar | 586 | 37.1 | 478 | 32.3 | 450 | 30.6 |  |
|  | < 40 hours | 505 | 35.4 | 322 | 25.4 | 527 | 39.2 | .0001 |
| Working hours  (per week) | 40~52 hours | 412 | 22.7 | 809 | 43.4 | 632 | 33.9 |  |
|  | ≥ 52 hours | 195 | 26.3 | 347 | 46.8 | 199 | 26.9 |  |
| Employment status | Full-time | 575 | 21.6 | 1123 | 44.1 | 915 | 34.3 | .0001 |
|  | Temporary/Daily | 537 | 39.5 | 355 | 26.3 | 443 | 34.2 |  |
|  | Day work | 871 | 26.2 | 1307 | 40.6 | 1102 | 33.3 | .0001 |
| Work schedule | Rotational shift work | 67 | 26.8 | 69 | 35.0 | 83 | 38.2 |  |
|  | Other | 174 | 37.8 | 102 | 23.0 | 173 | 39.2 |  |
| Sum | | 1112 | 27.6 | 1478 | 38.2 | 1358 | 34.2 |  |
