# Supplementary material for: Association between long working hours and unmet dental needs in wage workers
Source: BMC Oral Health. 2023 Aug 13;23:570. doi: 10.1186/s12903-023-03289-0 (PMC10424332; doi:10.1186/s12903-023-03289-0)
Supplement: Supplementary file 2 — Additional file 2. Distribution of man study subjects by working hours. [file 12903_2023_3289_MOESM2_ESM.docx]

Additional file 2: Distribution of man study subjects by working hours.

|  | | Total | < 40 hours |  | 40~52 hours | | ≥ 52 hours | |  |
| --- | --- | --- | --- | --- | --- | --- | --- | --- | --- |
|  |  |  | N | wt% | N | wt% | N | wt% | *P* |
| Unmet dental needs | No | 4177 | 875 | 19.7 | 2325 | 56.7 | 977 | 23.6 |  |
|  |  |  |  |  |  |  |  |  | .000 |
|  | Yes | 1786 | 345 | 18.6 | 934 | 51.9 | 507 | 29.4 |  |
|  | Economic issues | 449 | 131 | 25.4 | 193 | 45.3 | 125 | 29.3 |  |
|  |  |  |  |  |  |  |  |  | .0001 |
|  | Lack of Time | 736 | 94 | 13.9 | 406 | 53.2 | 236 | 33.0 |  |
|  | Other | 601 | 120 | 19.7 | 335 | 55.2 | 146 | 25.1 |  |
|  | 20~39 | 2281 | 409 | 19.1 | 1305 | 56.1 | 567 | 24.8 |  |
|  |  |  |  |  |  |  |  |  | .0001 |
| Age group  (y) | 40~59 | 2713 | 400 | 14.4 | 1,653 | 60.0 | 660 | 25.6 |  |
|  | ≥ 60 | 969 | 411 | 39.9 | 301 | 33.3 | 257 | 26.8 |  |
|  | Experience | 4733 | 882 | 16.6 | 2632 | 56.8 | 1219 | 26.6 |  |
|  |  |  |  |  |  |  |  |  | .0001 |
| Marital status |  |  |  |  |  |  |  |  |  |
|  | In-Experience | 1230 | 338 | 27.3 | 627 | 50.9 | 265 | 21.8 |  |
|  | ≤ Middle school | 873 | 296 | 29.7 | 308 | 37.2 | 269 | 33.0 |  |
|  |  |  |  |  |  |  |  |  | .0001 |
| Education  level | High school | 2079 | 502 | 24.3 | 1000 | 48.5 | 577 | 27.3 |  |
|  | ≥ University | 3011 | 422 | 13.3 | 1951 | 64.6 | 638 | 22.1 |  |
|  | Under | 399 | 224 | 51.4 | 100 | 27.5 | 75 | 21.1 |  |
|  |  |  |  |  |  |  |  |  | .0001 |
| Household income | Medium Low | 1364 | 322 | 23.5 | 622 | 45.6 | 420 | 30.9 |  |
|  | Slander | 1980 | 315 | 14.9 | 1134 | 58.0 | 531 | 27.0 |  |
|  | Award | 2220 | 359 | 15.8 | 1403 | 63.0 | 458 | 21.1 |  |
|  | Dong | 5075 | 1032 | 19.5 | 2808 | 55.9 | 1235 | 24.6 |  |
|  |  |  |  |  |  |  |  |  | .046 |
| Residence |  |  |  |  |  |  |  |  |  |
|  | Eup, Myeon | 888 | 188 | 18.7 | 451 | 51.6 | 249 | 29.8 |  |
|  | White collar | 2830 | 480 | 16.4 | 1860 | 65.8 | 490 | 17.8 |  |
|  |  |  |  |  |  |  |  |  | .0001 |
| Occupational  group | Pink collar | 633 | 178 | 30.2 | 278 | 43.2 | 177 | 26.6 |  |
|  | Blue collar | 2500 | 562 | 19.7 | 1121 | 46.6 | 817 | 33.7 |  |
|  | Full-time | 4643 | 559 | 11.2 | 2843 | 61.5 | 1241 | 27.3 |  |
| Working hours  (per week) |  |  |  |  |  |  |  |  | .0001 |
|  | Temporary/Daily | 1,320 | 661 | 47.6 | 416 | 34.0 | 243 | 18.5 |  |
|  | Day work | 4881 | 938 | 17.3 | 2866 | 59.4 | 1,077 | 23.3 |  |
|  |  |  |  |  |  |  |  |  | .0001 |
| Work schedule | Rotational shift work | 597 | 52 | 9.2 | 258 | 46.2 | 287 | 44.7 |  |
|  | Other | 485 | 230 | 48.7 | 135 | 28.0 | 120 | 23.3 |  |
| Sum | | 5963 | 1220 | 19.4 | 3259 | 55.3 | 1484 | 25.4 |  |
