# Supplementary material for: Association between long working hours and unmet dental needs in wage workers
Source: BMC Oral Health. 2023 Aug 13;23:570. doi: 10.1186/s12903-023-03289-0 (PMC10424332; doi:10.1186/s12903-023-03289-0)
Supplement: Supplementary file 3 — Additional file 3. Distribution of woman study subjects by working hours. [file 12903_2023_3289_MOESM3_ESM.docx]

Additional file 3: Distribution of woman study subjects by working hours.

|  | | Total | < 40 hours |  | 40~52 hours | | ≥ 52 hours |  |  |
| --- | --- | --- | --- | --- | --- | --- | --- | --- | --- |
|  |  |  | N | wt% | N | wt% | N | wt% | *P* |
| Unmet dental needs | No | 3979 | 1895 | 47.4 | 1697 | 43.3 | 387 | 9.3 |  |
|  |  |  |  |  |  |  |  |  | .423 |
|  | Yes | 2162 | 1009 | 46.6 | 919 | 42.9 | 234 | 10.5 |  |
|  | Economic burdens | 663 | 374 | 55.2 | 219 | 33.9 | 70 | 10.9 |  |
|  |  |  |  |  |  |  |  |  | .0001 |
|  | Lack of time | 742 | 228 | 31.3 | 403 | 54.7 | 111 | 14.1 |  |
|  | Other | 757 | 407 | 54.4 | 297 | 39.0 | 53 | 6.6 |  |
|  | 20~39 | 2365 | 910 | 38.8 | 1262 | 52.7 | 193 | 8.5 |  |
|  |  |  |  |  |  |  |  |  | .0001 |
| Age group  (y) | 40~59 | 2769 | 1285 | 47.9 | 1157 | 41.1 | 327 | 11.0 |  |
|  | ≥ 60 | 1007 | 709 | 71.2 | 197 | 19.5 | 101 | 9.3 |  |
|  | Experience | 4808 | 2408 | 50.3 | 1904 | 39.7 | 496 | 10.0 |  |
|  |  |  |  |  |  |  |  |  | .0001 |
| Marital status |  |  |  |  |  |  |  |  |  |
|  | In-Experience | 1333 | 496 | 37.8 | 712 | 53.2 | 125 | 9.0 |  |
|  | ≤ Middle school | 1521 | 930 | 61.1 | 376 | 25.0 | 215 | 14.0 |  |
|  |  |  |  |  |  |  |  |  | .0001 |
| Education  level | High school | 2116 | 1008 | 49.2 | 869 | 40.0 | 239 | 10.8 |  |
|  | ≥ University | 2504 | 966 | 38.4 | 1371 | 55.0 | 167 | 6.7 |  |
|  | Under | 738 | 547 | 75.2 | 140 | 19.6 | 51 | 5.2 |  |
|  |  |  |  |  |  |  |  |  | .0001 |
| Household income | Medium low | 1481 | 746 | 50.4 | 557 | 38.3 | 178 | 11.3 |  |
|  | Slander | 1852 | 790 | 42.9 | 858 | 46.2 | 204 | 10.8 |  |
|  | Award | 2070 | 821 | 39.4 | 1061 | 51.5 | 188 | 9.2 |  |
|  | Dong | 5249 | 2470 | 46.7 | 2257 | 43.7 | 522 | 9.6 |  |
|  |  |  |  |  |  |  |  |  | .292 |
| Residence |  |  |  |  |  |  |  |  |  |
|  | Eup, Myeon | 892 | 434 | 49.6 | 359 | 39.9 | 99 | 10.5 |  |
|  | White collar | 2850 | 1059 | 37.3 | 1629 | 57.0 | 162 | 5.7 |  |
|  |  |  |  |  |  |  |  |  | .0001 |
| Occupational  group | Pink collar | 1460 | 815 | 58.0 | 425 | 28.8 | 220 | 13.2 |  |
|  | Blue collar | 1831 | 1030 | 54.3 | 562 | 32.2 | 239 | 13.5 |  |
|  | Full-time | 3786 | 1241 | 32.5 | 2151 | 57.2 | 394 | 10.3 |  |
| Working hours  (per week) |  |  |  |  |  |  |  |  | .0001 |
|  | Temporary/Daily | 2355 | 1663 | 71.0 | 465 | 20.1 | 227 | 8.8 |  |
|  | Day work | 5131 | 2269 | 43.7 | 2359 | 46.9 | 503 | 9.4 |  |
|  |  |  |  |  |  |  |  |  | .0001 |
| Work schedule | Rotational shift | 187 | 43 | 25.4 | 103 | 54.2 | 41 | 20.3 |  |
|  | Other | 823 | 592 | 72.3 | 154 | 18.5 | 77 | 9.3 |  |
| Sum | | 6141 | 2904 | 47.1 | 2616 | 43.2 | 621 | 9.7 |  |
